# Prediction of postoperative pulmonary complications using preoperative controlling nutritional status (CONUT) score in patients with resectable non-small cell lung cancer

**DOI:** 10.1038/s41598-020-68929-9

**Published:** 2020-07-24

**Authors:** Sang Chul Lee, Jin Gu Lee, Sang Hoon Lee, Eun Young Kim, Joon Chang, Dae Joon Kim, Hyo Chae Paik, Kyung Young Chung, Ji Ye Jung

**Affiliations:** 1 Division of Pulmonology, Department of Internal Medicine, Severance Hospital, Yonsei University College of Medicine, Seoul, Republic of Korea; 2 Department of Thoracic and Cardiovascular Surgery, Severance Hospital, Yonsei University College of Medicine, Seoul, Republic of Korea; 3 Division of Pulmonology, Department of Internal Medicine, National Health Insurance Service Ilsan Hospital, Goyang, Republic of Korea

**Keywords:** Non-small-cell lung cancer, Surgical oncology

## Abstract

Postoperative pulmonary complications (PPCs) significantly impact surgical outcome. We investigated the predictive ability of controlling nutritional status (CONUT) for PPC after lung resection in patients with non-small cell lung cancer (NSCLC). We retrospectively reviewed data of 922 patients with NSCLC who underwent complete resection from January 2016–December 2017. We analyzed the frequency and characteristics of PPCs and compared receiver operating characteristic (ROC) curves of various prognostic models to predict PPCs. A CONUT score higher than 1 was considered as a high CONUT score. Total incidence of PPCs was 8.6% (n = 79). The proportion of pneumonia was significantly larger in the high CONUT group (*P* < 0.05). The CONUT consistently had a higher area under curve (AUC) value (0.64) than other prognostic models (prognostic nutritional index (PNI): AUC = 0.61, Glasgow prognostic score (GPS): AUC = 0.57, and assessment of respiratory risk in surgical patients in Catalonia (ARISCAT): AUC = 0.54). Multivariate analysis identified underweight [Odds ratio (OR) = 4.57, *P* = 0.002] and high CONUT score (OR = 1.91, *P* = 0.009) as independent PPCs prognostic factors. One-year mortality rate for high CONUT score was significantly higher (hazard ratio = 7.97; 95% confidence interval, 1.78–35.59). Preoperative CONUT score is an independent predictor of PPCs and 1-year mortality in patients with resectable NSCLC.

## Introduction

Postoperative complication (PPC) is critically important for patient outcomes, and is associated with mortality, morbidity, and length of stay in both thoracic and non-thoracic surgical patients^1^. Although the definition of PPCs varies in the literature, it generally includes prolonged air leak, pneumothorax, atelectasis, pleural effusion, respiratory infections, broncho-pleural fistula, and acute respiratory distress syndrome (ARDS)^2^.

In patients undergoing thoracic surgery in particular, the incidence of PPCs is reported to be between 3 and 49%, with mortality ranges ranging 2–12%^3^. Therefore, careful preoperative evaluation is needed to predict PPCs, and this is a common concern among thoracic surgeons, pulmonologists, and anesthesiologists. Various PPCs prediction models have been previously developed; these include the assessment of respiratory risk in surgical patients in Catalonia (ARISCAT) model, Gupta risk calculators, and the respiratory failure risk index suggested by Arozullah et al.^2,4–6^. However, these models require diverse preoperative parameters, which bring questionable clinical usefulness of those. Moreover, detailed nutritional status is not reflected in any of these models.

Recently, a number of studies have reported that the presence of a systemic inflammatory response and malnutrition is associated with poor prognosis in various malignancies. It has also been shown that systemic inflammation-based prognostic scores, such as the prognostic nutritional index (PNI) and Glasgow prognostic score (GPS), have independent prognostic value regardless of tumor stage in various malignancies, including non-small cell lung cancer (NSCLC)^7–11^.

Similar to these inflammation-based prognostic scores, the controlling nutritional status (CONUT) score, which is calculated using serum albumin, total cholesterol, and total peripheral lymphocyte count, was suggested as a screening tool for early detection of under-nutrition^12^. The CONUT scoring tool is low-cost and simple to use. Previous studies have demonstrated its relationship with long-term clinical outcomes in various malignancies^13–18^.

However, the clinical significance of the CONUT score for predicting postoperative outcomes in patients with operable NSCLC who have undergone lung resection is still unknown. Therefore, this study aimed to investigate whether the CONUT score can serve as an independent predictor of PPCs in patients with completely resected NSCLC.

## Materials and methods

### Study population and data collection

We conducted this retrospective observational study using the data of 934 patients who underwent lung resection surgery with curative intent for resectable NSCLC at Severance Hospital (Seoul, South Korea) between January 2016 and December 2017. Of these, 12 patients were excluded owing to unmeasured laboratory data, surgically confirmed advanced stage (M1), or other insufficient data (Supplementary Fig. 1). Preoperative and postoperative clinical data were collected from the medical records and database. Laboratory data were obtained within 1 week before the surgery. The Institutional Review Board of Severance Hospital approved the study protocol and waived the informed consent from the patients due to the retrospective nature of the study (IRB No.: 4-2018-0922).

### Study design

Preoperative and postoperative clinical characteristics, PPCs, and 1-year overall mortality were compared between high and low CONUT groups. The ability of the CONUT score to predict major PPCs was analyzed and compared with that of the PNI, GPS and ARISCAT.

### Definitions

PPCs were defined as any one or more of the following complications: prolonged air leak, pneumonia, pneumothorax, chylothorax, ARDS, bleeding, bronchopulmonary fistula, empyema, atelectasis, and pleural effusion^1^. Bleeding complication was defined as reoperation or transfusion of three or more red blood cell packs to control massive hemoptysis or continuous bleeding through the chest tube. Respiratory infection within 1 month before surgery was defined as recent respiratory infection. Low preoperative SpO_2_ was defined as below 96% and anemia was defined as hemoglobin level below 10.0 g/dL. Obesity and underweight were defined as body mass index (BMI) ≥ 25 kg/m^2^ and BMI < 18.5 kg/ m^2^, respectively.

Table 1 shows inflammation-based prognostic scores and postoperative pulmonary risk scoring system, which were used in our study. The CONUT score (range, 0–12) was assessed, using serum albumin level, total lymphocyte count, and total cholesterol level. The ARISCAT risk score was calculated as the sum of seven patient- or surgery-related risk factor scores (range, 0–123). PNI was calculated using serum albumin level and total lymphocyte count and was scored as 0 or 1. GPS was calculated using serum C-reactive protein and albumin levels and was also scored as 0 or 1.

**Table 1:** Inflammation-based prognostic scores and postoperative pulmonary risk scoring system.

	**Parameters**	**Score**			
**CONUT**	1. Albumin	≥ 3.50 (0)	3.00–3.49 (2)	2.50–2.99 (4)	< 2.50 (6)
	2. Total lymphocyte count, /mL	> 1,600 (0)	1,200–1599 (1)	800–1,199 (2)	< 800 (3)
	3. Total cholesterol, mg/dL	> 180 (0)	140–180 (1)	100–139 (2)	< 100 (3)
	Assessment (1 + 2 + 3)	Normal (0–1)	Mild (2–4)	Moderate (5–8)	Severe (9–12)
**PNI**	**Calculation**	**Score**			
	Albumin (g/L) + 5 × total lymphocyte count/mL ≥ 45	0			
	Albumin (g/L) + 5 × total lymphocyte count/mL < 45	1			
**GPS**	**Calculation**	**Score**			
	C-reactive protein ≤ 10 mg/L and albumin ≥ 35 g/L	0			
	C-reactive protein ≤ 10 mg/L and albumin < 35 g/L	1			
	C-reactive protein > 10 mg/L and albumin ≥ 35 g/L	1			
	C-reactive protein > 10 mg/L and albumin < 35 g/L	2			
ARISCAT*	**Risk factors**	**Risk score**			
	Age, year	≤ 50 (0)	51–80 (3)	> 80 (16)	
	Preoperative SpO_2_, %	≥ 96 (0)	91–95 (8)	≤ 90 (24)	
	Respiratory infection in last month	No (0)	–	Yes (17)	
	Preoperative Hb ≤ 10 g/dL	No (0)	–	Yes (11)	
	Surgical incision site	Peripheral (0)	Upper abdominal (15)	Intrathoracic (24)	
	Duration of surgery, hours	< 2 (0)	2–3 (16)	> 3 (23)	
	Emergency procedure	No (0)	–	Yes (8)	
	Risk class*	**No. of points in risk score (pulmonary complication rate)**			
	Low	< 26 (1.6%)			
	Intermediate	26–44 (13.3%)			
	High	< 44 (42.1%)			

*ARISCAT risk class is determined by sum of each score of risk factors.

*CONUT* controlling nutritional status, *PNI* Prognostic nutritional index, *GPS* Glasgow prognostic score, *ARISCAT* The Assess Respiratory Risk in Surgical Patients in Catalonia Risk Index: Independent Predictors of Postoperative Pulmonary Complications, *SpO*_*2*_ oxygen saturation, *Hb* hemoglobin.

**Figure 1:**
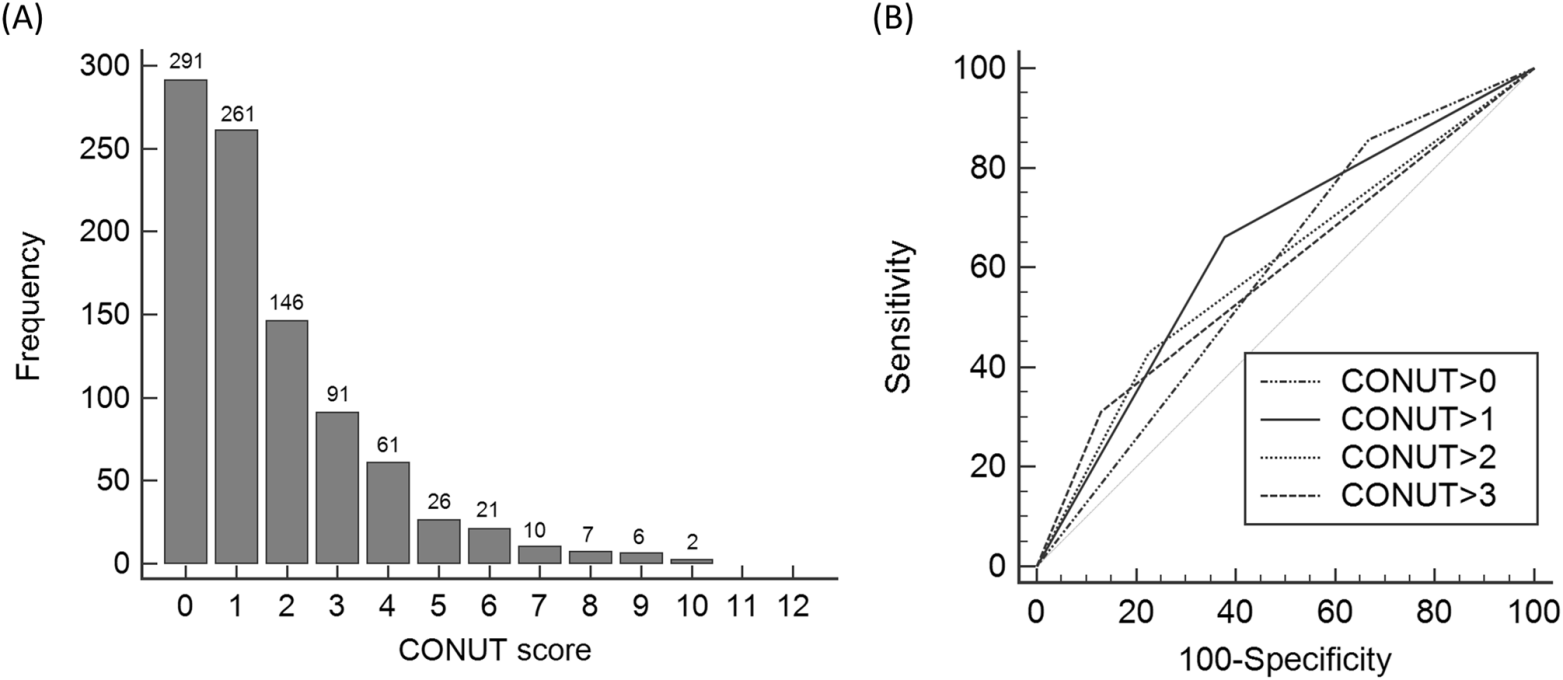
Frequency distribution of CONUT scores and comparison of the area under the ROC curve for PPCs predictability of the CONUT depending on different cut-off values. (**A**) Distribution of the study population depending on CONUT score. Approximately half of the study population had a CONUT score of 0 or 1 (452 of 922, 49.0%). (**B**) ROC curves for PPCs according to the various cut-off values of CONUT. The AUCs were 0.594 (CONUT > 0), 0.642 (CONUT > 1), 0.601 (CONUT > 2), and 0.591 (CONUT > 3). *AUC* area under curve, *CONUT* controlling nutritional status, *PPCs* postoperative pulmonary complications, *ROC* receiver operating characteristic, *CI* confidence interval.

The optimal cut-off values for CONUT, PNI, GPS and ARISCAT scores were determined by receiver operating characteristic (ROC) curves. The best cut-off points for CONUT, PNI, GPS, ARISCAT score were 1.0, 43.4, 1.0, and 49.0, respectively, corresponding to maximum joint sensitivity and specificity. Distribution of study population depending on CONUT scores and the comparison of ROC curves between the different cut-off values of CONUT are described in Fig. 1A and B, respectively. Those with a CONUT score > 1.0 were categorized into the high CONUT group while those with score of ≤ 1.0 were categorized into the low CONUT group.

### Statistical analysis

We conducted statistical comparison using SPSS Statistics Version 23 (IBM Co., Armonk, NY, USA). We conducted univariate analyses with χ^2^ test for categorical variables and Student’s t-test for continuous variables. Significant prognostic factors for PPCs in univariate analyses were selected for multivariate logistic analysis. We evaluated the ability of CONUT, PNI, GPS, and ARISCAT scores to predict PPCs using the ROC curve. We compared 1-year overall mortality between high and low CONUT groups through the Kaplan–Meier method and conducted Cox regression analyses to determine independent prognostic factors. A value of *P* < 0.05 was considered to be statistically significant.

### Ethical standards

This article does not contain any studies with human or animal subjects performed by any of the authors.

## Results

### Baseline characteristics

A total of 922 patients were eligible to be included in this study. Patient characteristics and clinic-pathologic features are presented in Tables 2 and 3; the mean patient age was 64.2 years and 522 (56.6%) of the patients were men. Mean follow-up duration was 20.4 months. The majority of patients were diagnosed with stage I NSCLC (n = 665, 72.1%), and the most common histologic type was adenocarcinoma (n = 715, 77.5%). The most common type of operation was lobectomy (80.8%), followed by segmentectomy (6.9%), wedge resection (5.6%), bilobectomy (3.5%), and pneumonectomy (3.2%). Eighty-five percent of the patients underwent video-assisted thoracoscopic surgery.

**Table 2:** Patient characteristics [Significant differences (p < 0.05)].

Characteristics	Total (n = 922)	CONUT > 1.0 (n = 370)	CONUT ≤ 1.0 (n = 552)	*p*-value
Age, > 65 years	485 (52.6)	235 (63.5)	250 (45.3)	< 0.001
Sex, man	522 (56.6)	235 (63.5)	287 (52.0)	< 0.001
Body mass index	24.1 ± 3.1	22.6 ± 1.7	24.3 ± 2.2	0.713
Obese (> 25 kg/m^2^)	337 (36.6)	121 (32.7)	216 (39.1)	0.051
Underweight (< 18.5 kg/m^2^)	23 (2.5)	13 (3.5)	10 (1.8)	0.131
Ever smoker	392 (42.5)	195 (52.7)	197 (35.5)	< 0.001
**Comorbidities**				
Hypertension	412 (44.7)	206 (55.7)	206 (37.3)	< 0.001
Diabetes	163 (17.7)	103 (27.8)	60 (10.9)	< 0.001
Chronic kidney disease	94 (10.2)	56 (15.1)	38 (6.9)	< 0.001
COPD	93 (10.1)	51 (13.8)	42 (7.6)	0.003
**Preoperative findings**				
Respiratory infection(within 1 month)	370 (40.1)	12 (3.2)	4 (0.7)	0.008
Hypoxemia (SpO_2_ < 96%)	15 (1.6)	10 (2.7)	5 (0.9)	0.059
**Laboratory tests**				
White blood cell count, /μL	6,633 ± 2,616	6,770 ± 3,535	6,541 ± 1745	< 0.001
Hemoglobin, g/dL	13.2 ± 1.4	12,8 ± 1.4	13.5 ± 1.3	0.128
Lymphocyte count, /μL	2069 ± 701	1816 ± 741	2,239 ± 617	< 0.001
Albumin, g/dL	3.7 ± 0.4	3.4 ± 0.5	3.8 ± 0.2	< 0.001
Cholesterol, mg/dL	175 ± 39	150 ± 37	192 ± 31	< 0.001
C-reactive protein, mg/dL	48.7 ± 38.2	52.8 ± 45.1	40.8 ± 31.9	< 0.001
**Pulmonary function**				
FEV_1_, L	2.3 ± 0.6	2.1 ± 0.5	2.3 ± 0.7	0.015
FEV_1_, %	99.1 ± 19.0	83.9 ± 15.2	94.3 ± 13.9	0.112
FEV_1_/FVC, %	0.7 ± 9.5	68.1 ± 11.6	65.9 ± 10.2	0.034
DLco, %	95.2 ± 18.3	83.7 ± 21.5	87.1 ± 12.2	0.008

*CONUT* controlling nutritional status, *COPD* chronic obstructive pulmonary disease, *DL*_*CO*_ diffusing capacity of the lungs for carbon monoxide, *FEV*_*1*_ forced expiratory volume, *FVC* forced vital capacity.

**Table 3:** Comparison of clinico-pathological features between high and low CONUT groups [Significant differences (p < 0.05)].

Clinical parameters	Total (n = 922)	CONUT > 1.0 (n = 370)	CONUT ≤ 1.0 (n = 552)	*P*-value
**Types of operation**				
Pneumonectomy	29 (3.2)	17 (4.6)	12 (2.2)	0.039
Lobectomy	745 (80.8)	291 (78.6)	454 (82.3)	0.174
Bilobectomy	32 (3.5)	19 (5.1)	13 (2.4)	0.024
Segmentectomy	64 (6.9)	25 (6.8)	39 (7.0)	0.857
Wedge resection	52 (5.6)	18 (4.9)	34 (6.1)	0.404
VATS	789 (85.6)	289 (78.1)	500 (90.6)	< 0.001
**Postoperative findings**				
p-stage				< 0.001
I	665 (72.1)	230 (62.1)	435 (78.8)	
II/III	257 (27.8)^†^	140 (37.8)**	117 (62.1)^††^	
Adenocarcinoma	715 (77.5)	248 (67.0)	467 (84.6)	< 0.001
**PPC predictors**				
CONUT score	1.6 ± 1.8	3.4 ± 1.7	0.5 ± 0.5	
PNI	42.6 ± 5.7	46.4 ± 5.9	40.1 ± 4.0	< 0.001
GPS	1.0 ± 0.6	1.4 ± 0.6	0.8 ± 0.4	< 0.001
ARISCAT	44.1 ± 3.9	45.0 ± 4.8	43.5 ± 3.0	< 0.001

*CONUT* controlling nutritional status, *PNI* prognostic nutritional index, *GPS* Glasgow prognostic score, *ARISCAT* assessment of respiratory risk in surgical patients in Catalonia, *VATS* video-assisted thoracoscopic surgery.

^†^In the II/III group, 144 (15.6%) and 13 (12.3%) patients were categorized as stage II and III, respectively.

**In the II/III group, 85 (23.0%) and 59 (14.9%) patients were categorized as stage II and III, respectively.

^††^In the II/III group, 59 (10.7%) and 58 (10.5%) patients were categorized as stage II and III, respectively.

Of the 922 patients, 370 (40.1%) and 552 (59.9%) patients were categorized into the high and low CONUT group, respectively. Compared to the low CONUT group, the high CONUT group had an older mean age and a larger proportion of patients who were men, smokers, and had various comorbidities, including hypertension, diabetes, chronic kidney disease, and chronic obstructive pulmonary disease (COPD). Among the pulmonary function parameters, forced expiratory volume in 1 s (FEV_1_) and diffusing capacity of the lungs for carbon monoxide (DLco) were lower in the high CONUT group than in the low CONUT group (Table 2). Pneumonectomy and bilobectomy were more frequently performed in the high CONUT group than in the low CONUT group. The low CONUT group had a higher proportion of patients with stage 1 NSCLC than the high CONUT group. The PNI, GPS, and ARISCAT scores were all significantly higher in the high CONUT group than in the low CONUT group (Table 3).

### Postoperative pulmonary complications in the high and low CONUT groups

PPCs between the high and low CONUT groups are compared in Table 4. Seventy-nine patients (8.6%) developed a total of 106 PPCs. Prolonged air leak (44.3%) was the most common PPC, followed by pneumonia (32.9%) and pneumothorax (11.3%). A higher frequency of total PPCs (12.7% vs. 5.8%, *P* < 0.001) was observed in the high CONUT group. Among all PPCs, prolonged air leak (5.4% vs. 2.7%, *P* = 0.036), pneumonia (4.6% vs. 1.6%, *P* < 0.008), and postoperative bleeding (1.4% vs. 0.2%, *P* = 0.041) were more frequently observed in the high CONUT group than in the low CONUT group.

**Table 4:** Postoperative pulmonary complication between high and low CONUT groups [Significant differences (p < 0.05)].

Postoperative pulmonary complication	Total (n = 922)	High CONUT (n = 370)	Low CONUT (n = 552)	*p*-value
Total	79 (8.6)	47 (12.7)	32 (5.8)	< 0.001
Prolonged air leak	35 (3.8)	20 (5.4)	15 (2.7)	0.036
Pneumonia	26 (2.8)	17 (4.6)	9 (1.6)	0.008
Pneumothorax	9 (1.0)	6 (1.6)	3 (0.5)	0.103
Chylothorax	8 (0.9)	4 (1.1)	4 (0.7)	0.567
ARDS	7 (0.8)	5 (1.4)	2 (0.4)	0.090
Bleeding	6 (0.7)	5 (1.4)	1 (0.2)	0.030
Bronchopulmonary fistula	5 (0.5)	3 (0.8)	2 (0.4)	0.183
Empyema	4 (0.4)	1 (0.3)	3 (0.5)	0.536
Atelectasis	3 (0.3)	2 (0.5)	1 (0.2)	0.348
Pleural effusion	3 (0.3)	2 (0.5)	1 (0.2)	0.348

*ARDS* acute respiratory distress syndrome, *CONUT* controlling nutritional status.

### Comparison between preoperative risk assessment scores for PPC predictability

**Figure 2:**
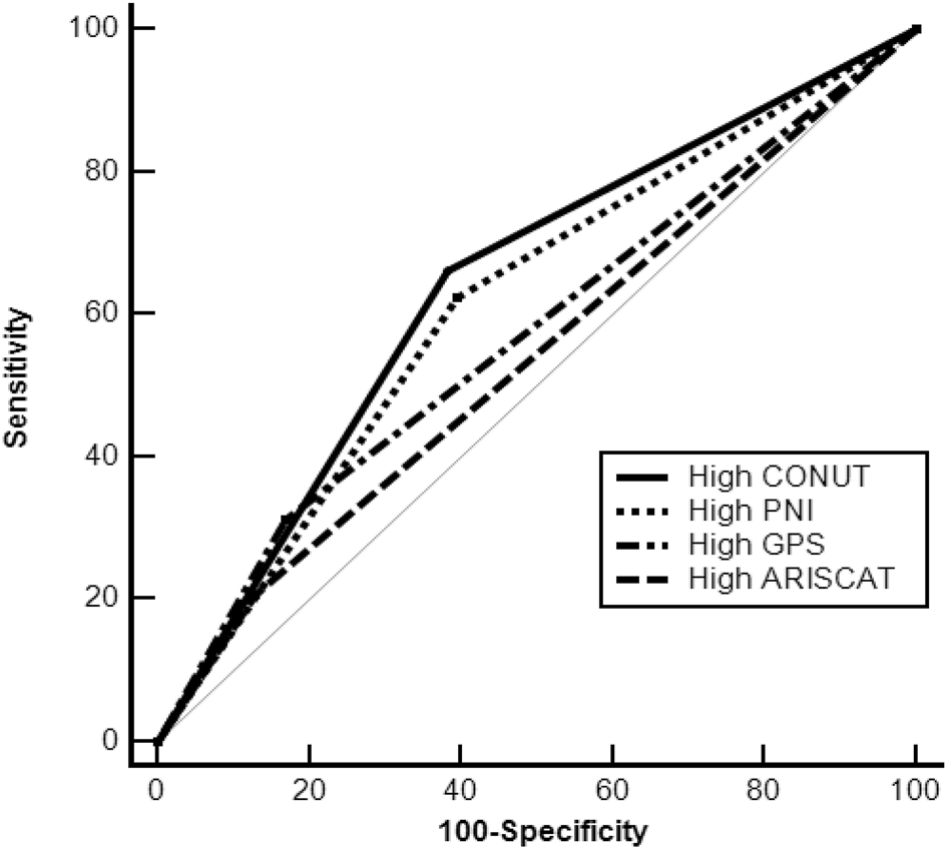
Comparison of the area under the ROC curve for PPCs predictability of the CONUT and inflammation-based prognostic scores/ARISCAT score. The ability of various preoperative risk assessment scores to predict PPCs was compared using ROC curve. The AUC of the CONUT, PNI, GPS, and ARISCAT were 0.64 (95% CI 0.63–0.69), 0.61 (95% CI 0.58–0.65), 0.57 (95% CI; 0.54–0.60) and 0.54 (95% CI 0.51–0.57), respectively. The AUC of the CONUT was significantly higher than that of the GPS (*P* = 0.01) and the ARISCAT (*P* < 0.01). *ARISCAT* assessment of respiratory risk in surgical patients in Catalonia, *AUC* are under curve, *CONUT* controlling nutritional status, *GPS* Glasgow prognostic score, *PNI* prognostic nutritional index, *PPC*s postoperative pulmonary complications, *ROC* receiver operating characteristic, *CI* confidence interval.

The ability of the four preoperative risk assessment scores to predict PPCs is shown in Fig. 2. The area under the curve (AUC) of CONUT, PNI, GPS, and ARISCAT was 0.64 (95% CI 0.63–0.69), 0.61 (95% CI 0.58–0.65), 0.57 (95% CI 0.54–0.60) and 0.54 (95% CI 0.51–0.57), respectively. The AUC of CONUT was significantly higher than that of GPS (*P* = 0.01) and ARISCAT (*P* < 0.01), while AUC of CONUT was non-significantly higher than that of PNI (*P* = 0.397).

According to the multivariate analysis of the risk factors for PPCs, high CONUT score had an odds ratio of 1.91 (95% CI, 1.17–3.10). Underweight was also an independent risk factor for PPCs (OR = 4.57; 95% CI, 1.76–11.83) (Table 5).

**Table 5:** Multivariate analyses of risk factors for postoperative pulmonary complications [Significant differences (p < 0.05)].

Variable	Univariate analysis		Multivariate analysis	
	Unadjusted OR (95% CI)	*p*-value	Adjusted OR (95% CI)	*p*-value
Age > 65 years	0.71 (0.45–1.14)	0.197	–	–
Sex, man	2.65 (1.55–4.51)	< 0.001	1.90 (0.93–3.89)	0.077
Underweight (BMI < 18.5 kg/m^2^)	6.12 (2.51–14.93)	< 0.001	4.57 (1.76–11.83)	0.002
Ever smoker	2.43 (1.51–3.91)	< 0.001	1.44 (0.76–2.72)	0.259
Recent respiratory infection (within 1 month)	2.48 (0.69–8.90)	0.155	–	–
Preoperative hypoxemia (SpO_2_ < 96%)	1.63 (0.36–7.37)	0.379	–	–
Preoperative anemia (Hb ≤ 10 g/dL)	0.74 (0.09–5.76)	1.000	–	–
p-Stage II/III vs. I	1.53 (0.94–2.50)	0.083	–	–
High CONUT (> 1)	2.42 (1.51–3.87)	< 0.001	1.91 (1.17–3.10)	0.009

*CI* confidence interval, *CONUT* controlling nutritional status, *HR* hazard ratio, *OR* odds ratio, *PPC* postoperative pulmonary complication(s).

### Relationship between preoperative nutritional status and postoperative mortality

**Figure 3:**
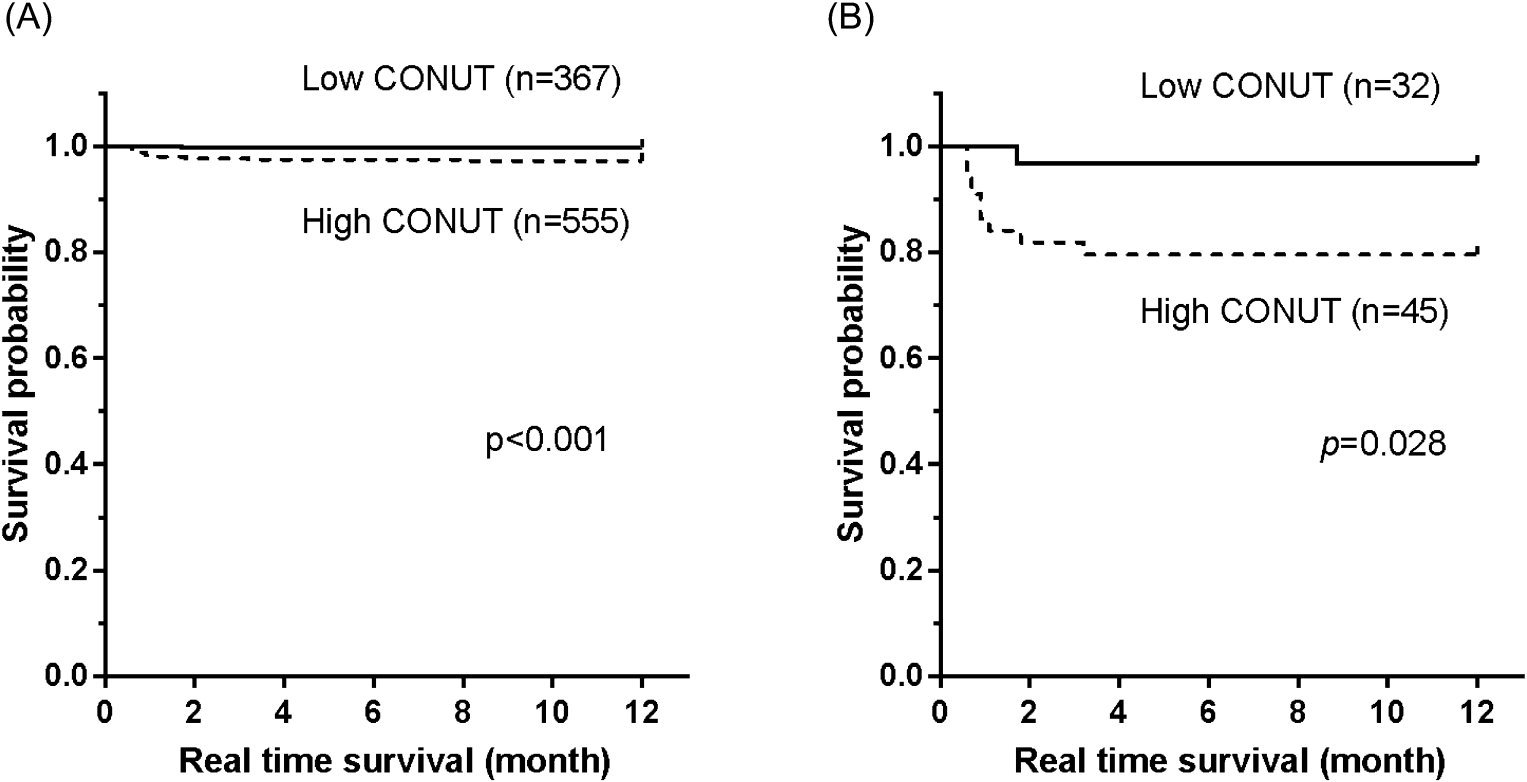
Kaplan–Meier survival curves according to CONUT status of (**A**) all subjects, (**B**) subjects with PPCs. One-year mortality was higher in the high CONUT group than in the low CONUT group, both in whole study population (*P* < 0.001; **A**) and among the 79 patients with PPCs (*P* = 0.028; **B**). *CONUT* controlling nutritional status, *PPCs* postoperative pulmonary complications

We analyzed the relationship between CONUT status and 1-year mortality; we found that 1-year mortality was higher in the high CONUT group compared to the low CONUT group (15 of 555 [2.7%] vs. 4 of 367 [0.1%], *P* < 0.001). Kaplan–Meier analysis revealed a significantly higher 1-year mortality rate in the high CONUT group in both the entire study population (*P* < 0.001; Fig. 3A) and among the 79 patients with PPCs (*P* = 0.028; Fig. 3B). According to the multivariate Cox regression analysis for 1-year mortality, high CONUT score had a hazard ratio of 6.62 (95% CI 1.43–30.66) (Table 6). Man (HR = 6.47; 95% CI 1.09–38.24), advanced pathologic stage (stage II-III) (HR = 6.49; 95% CI 1.94–21.71), and recent respiratory infection (HR = 13.11; 95% CI 2.71–63.45) were also independent risk factors for 1-year mortality.

**Table 6:** Cox regression analyses of risk factors for 1-year mortality [Significant differences (p < 0.05)].

Variable	Univariate analysis		Multivariate analysis	
	Unadjusted HR (95% CI)	*p*-value	Adjusted HR (95% CI)	*p*-value
Age > 65 years	2.21 (0.77–6.33)	0.129	–	–
Sex, man	5.96 (1.35–26.23)	0.007	6.47 (1.09–38.24)	0.039
Underweight (BMI < 18.5 kg/m^2^)	0.97 (0.96–0.98)	0.513	–	–
Ever smoker	2.76 (1.03–7.44)	0.036	0.49 (0.14–1.68)	0.260
Recent respiratory infection (within 1 month)	14.52 (3.72–56.68)	< 0.001	13.11 (2.71–63.45)	0.001
Preoperative hypoxemia (SpO_2_ < 96%)	4.23 (0.52–34.35)	0.141	–	–
Preoperative anemia (Hb ≤ 10 g/dL)	3.92 (0.48–31.7)	0.166	–	–
p-Stage II/III vs. I	9.06 (2.92–28.0)	< 0.001	6.49 (1.94–21.71)	0.002
High CONUT (> 1)	11.65 (2.65–51.28)	< 0.001	6.62 (1.43–30.66)	0.016

*CI* confidence interval, *CONUT* controlling nutritional status, *HR* hazard ratio, *OR* odds ratio, *PPC* postoperative pulmonary complication(s).

## Discussion

The results of this study suggest that a high CONUT score is associated with occurrence of PPCs after lung resection in patients with NSCLC. Moreover, in these patients, a high CONUT score is a significant prognostic factor for 1-year mortality. The CONUT score has been previously reported as a useful prognostic predictor for various malignant tumors, but data on its use in case of completely resected NSCLC are limited^13–18^. Thus, this is the first study to investigate the usefulness of the CONUT score for predicting PPCs and 1-year mortality in patients with completely resected NSCLC. Among the patients who underwent lung resection, 8.6% developed PPCs; this is a much lower rate than previously reported in patients after thoracic surgery (19–59%)^19^. Stephan et al. reported a PPC rate of 25% following lung resection performed by open thoracotomy^3^. In our study, 85% of patients underwent video-assisted thoracoscopic surgery, which might have resulted in better clinical outcomes. We found that prolonged air leak was the most frequent PPC, followed by pneumonia; this was in accordance with the results previously reported by Stephan et al.^3^

However, the 1-year mortality rate in our study (2.0%) was lower than that reported in previous studies. Short-term prognosis of NSCLC patients that underwent lung resection varied depending on the follow-up period, type of surgical procedure, and characteristics of study populations. Veen et al. previously described operative mortality, which was defined as death at any time during initial hospitalization or within 30 days after surgery, and was 2.1% among patients who underwent pulmonary surgery due to NSCLC^20^. However, another study by Ren et al., which showed the short-term effects of thoracoscopic segmentectomy and thoracoscopic lobectomy on solitary pulmonary nodules and early-stage lung cancer, demonstrated no death among 82 patients within the 1-year follow-up period^21^. In contrast, Eguchi et al. reported a 1-year mortality rate of 4.1% in stage I non-small-cell lung cancer patients. The most common cause of death was non-cancer specific (50%), followed by lung cancer specific (27.8%), and other cancer specific (27.8%). Moreover, non-cancer specific mortality represents a significant competing event for lung cancer-specific mortality, with an increasing impact as age increases^22^. These studies suggest that the composition of the study population could affect short-term mortality rates. In our study, more than two-thirds of the study population had stage I cancer, and relatively young patients below 65 years of age accounted for more than half of the study population. This could be a possible reason why our study showed a lower short-term mortality rate.

Precise quantitative prediction of PPCs risk factors is essential for surgeons and anesthesiologists to prepare safe patient management plans. For decades, various risk factors have been identified to aid in predicting PPCs, and several risk scoring systems have been developed to comprehensively evaluate PPCs^2,19,23^. Among these, the ARISCAT score is simple to use in clinical practice and has been prospectively validated for use in estimating PPCs^24^. However, in this study, most of the study population was elderly patients with NSCLC who underwent lung resection, and they were thus classified in the same high-risk group. Therefore, a more strategic tool is needed to classify patients in a similar risk category who are more likely to develop PPCs. For this purpose, the CONUT score showed superiority in predicting PPCs, as compared with the other inflammatory prognostic marker (GPS) or preoperative risk scoring system (ARISCAT score); AUC analysis showed that the CONUT score was significantly superior to the GPS and the ARISCAT score for predicting PPCs. However, non-significant higher AUC was observed for CONUT compared to PNI, which could be attributable to common components such as albumin level and total lymphocyte counts included in both the tools.

The CONUT score is based on immune-nutritional parameters composed of three key laboratory findings: serum albumin concentration, total lymphocyte count, and serum cholesterol level^13^.

Serum albumin is widely used to assess nutritional status, as well as current status of systemic illness and inflammation^25^, although hypoalbuminemia is associated with an impaired immune response through macrophage activation, there is evidence that shows that hypoalbuminemia is also associated with older age, independent of elevated C-reactive protein (CRP) levels. Hypoalbuminemia may have additional prognostic value besides its relationship with systemic inflammatory response^26,27^. Accordingly, perioperative nutritional support is considered as an effective strategy to improve surgical outcomes. Even though routine supplemental administration of albumin showed no apparent advantages in the treatment of patients in the surgical ICU, findings regarding the effect of pre- or postoperative nutritional intervention, regardless of the route and formula used, were promising^28–30^. In addition, the study by Kabata et al. demonstrated that the preoperative nutritional support could be helpful to maintain proper nutritional status and to reduce the number and severity of postoperative complications even in non-malnourished patients^31^. The association between low serum albumin and risk of PPCs is well-supported in the literature^2,6,23,32^. Lymphocytopenia is also related with malnutrition and suppression of cellular immunity. Impaired cell-mediated immunity leads to a weakened antibacterial cellular immune response, contributing to an increased chance of bacterial infection^33,34^. Moreover, the immune response to tumors is lymphocyte-dependent, and as a result, a low count can be a predictor of poor survival. Regarding the role of serum cholesterol levels in the CONUT score, hypocholesterolemia is more significantly associated with fewer circulating lymphocytes, total T cells, and CD8+ cells than is hypercholesterolemia. Moreover, cholesterol increases the antigen-presenting function of monocytes^35^. Therefore, a low serum total cholesterol level may contribute to a poorer prognosis by affecting intracellular signaling and impairing the immune system against infection, wound healing, or tumor spread.

The association between chronic inflammation and the life-cycle of tumor cells (cellular transformation, survival, proliferation, invasion, and metastasis) is well known, and suggests that inflammatory markers could be prognostic factors in malignancies^36,37^. The CONUT score was previously reported to be an independent predictor of overall survival and relapse-free survival in patients with resectable thoracic esophageal squamous cell carcinoma and in those who undergo curative hepatectomy for hepatocellular carcinoma^13,18^. In patients with colorectal cancer, preoperative CONUT scores predicted survival and postoperative severe complications^14^. Previous studies have also revealed the prognostic predictability of CONUT score for patients with NSCLC: the CONUT score was an independent prognostic factor for disease-free and overall survival in patients with lung adenocarcinoma with obstructive lung disease^38^. However, the CONUT score was not found to be a significant predictive or prognostic factor for the surgical outcomes of elderly patients with NSCLC^39^. Our study demonstrated the usefulness of the CONUT score in predicting both PPCs and 1-year mortality in patients with NSCLC undergoing curative resection; these results provide important support for the use of the CONUT score in this patient population.

However, unlike CONUT, GPS and PNI are composed of two variables and are relatively easy to calculate. To measure GPS, albumin level and CRP level are needed. Although CRP level is well-correlated with systemic inflammation and could be used to detect SIRS progression, there could be false positive results in the early-phase of infection or in immunocompromised hosts^40,41^. In case of PNI, albumin and total cholesterol are used as variables to measure similar to the CONUT score. This could be why the AUCs of CONUT and PNI showed similar results. However, the CONUT score, which is different from PNI, includes total cholesterol as a variable. As noted above, hypocholesterolemia is known to reflect malnutrition and autoimmune disease in cancer patients. Hence, additional research including advanced NSCLC cases is needed to compare AUCs in terms of PPC predictability and survival rate.

The strength of our study is that the CONUT score is an easily available and cost-effective biomarker which can be combined with preexisting prognostic parameters, thus assisting decision making based on its prognostic value. In addition, immune-nutritional deficiency is a modifiable factor with promising data on a targeted preoperative nutritional supplementation that may decrease morbidity and improve survival of cancer patients^42–44^.

However, a few limitations of this study should be considered. First, this was a single-center study with a relatively small sample size compared to other multi-center studies. However, compared to previous studies on the CONUT score, the sample size of this study is relatively large, and comprises patients across 2 years in a single center. Moreover, a single-center study would represent a limited number of surgeons, which lessens the variation in medical management and surgical techniques. Second, this study is likely to have selection and analytical biases due to the retrospective design. However, of the 934 patients who underwent lung resection surgery for NSCLC during the study period, only 12 patients were excluded owing to insufficient data or advanced stage. Moreover, surgeons might have changed their care for patients who were in a malnourished state. However, regardless of nutritional status, surgeons educated all the patients planning to undergo lung resection due to lung cancer about nutritional support, respiration, and exercise in the same manner. Moreover, because surgical scheduling rapidly proceeded once the patients were diagnosed with lung cancer, there was only a short period of time to administer nutritional support before surgery. Third, the observation period after surgery was relatively short to evaluate long-term survival outcomes. Although the mean follow-up period in this study was sufficient to evaluate PPC outcomes, further studies are needed to investigate long-term outcomes with respect to preoperative inflammatory prognostic scores. Lastly, potential factors that may affect inflammation and nutritional markers in the CONUT score, such as medication and nutritional support, were not included in the analysis.

## Conclusions

The CONUT score was found to be an independent predictor of PPCs, and it was also found to be superior to the PNI, GPS, and ARISCAT in terms of predicting postoperative prognosis in patients with resectable NSCLC. The variables used to calculate the CONUT score are readily available from laboratory data in daily clinical practice and preoperative evaluation, and thus the CONUT score is low cost and feasible in the clinical setting. Our findings provide evidence that surgeons can identify patients who are at high risk for PPCs using the CONUT score, and can then tailor perioperative care and ultimately improve postoperative outcomes for patients with NSCLC who are undergoing curative lung resection. However, further studies will be needed to determine the effect of nourishment before surgery in patients with a low CONUT score.

## Supplementary information

Supplementary figure 1

Supplementary data
