# Supplementary material for: Prediction of postoperative pulmonary complications using preoperative controlling nutritional status (CONUT) score in patients with resectable non-small cell lung cancer
Source: Sci Rep. 2020 Jul 24;10:12385. doi: 10.1038/s41598-020-68929-9 (PMC7382444; doi:10.1038/s41598-020-68929-9)
Supplement: Supplementary file 1 — Supplementary figure 1 [file 41598_2020_68929_MOESM1_ESM.docx]

**Prediction of postoperative pulmonary complications using preoperative controlling nutritional status (CONUT) score in patients with resectable non-small cell lung cancer**

**Author names**

Sang Chul Lee^1,3^, Jin Gu Lee^2*^, Sang Hoon Lee^1^, Eun Young Kim^1^, Joon Chang^1^, Dae Joon Kim^2^, Hyo Chae Paik^2^, Kyung Young Chung^2^, and Ji Ye Jung^1*^

**Affiliations**

***^1^****Division of Pulmonology, Department of Internal Medicine, Severance Hospital, Yonsei University College of Medicine, Seoul, Republic of Korea*

*^2^Department of Thoracic and Cardiovascular Surgery, Severance Hospital, Yonsei University College of Medicine, Seoul, Republic of Korea*

*^3^Division of Pulmonology, Department of Internal Medicine, National Health Insurance Service Ilsan Hospital, Goyang, Republic of Korea*

* These authors contributed equally to this work as corresponding authors.

**Corresponding Author**

Ji Ye Jung, M.D., Ph.D.

Associate Professor

Division of Pulmonology, Department of Internal Medicine,

Severance Hospital, Yonsei University College of Medicine

50-1 Yonsei-ro, Seodaemun-gu, Seoul 03722, Republic of Korea.

Tel: 82-10-3141-2576. Fax: 82-2-2228-2273

E-mail: [stopyes@yuhs.ac](mailto:stopyes@yuhs.ac)

**
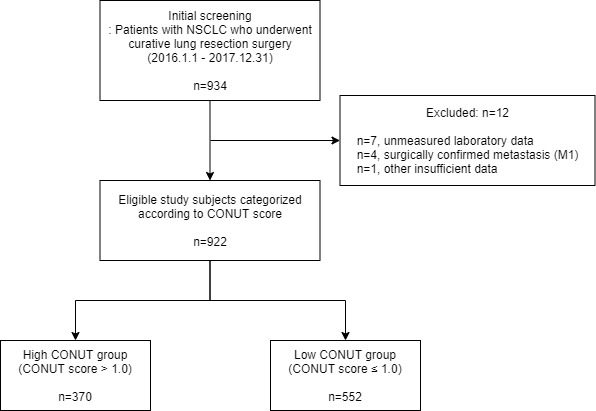
**

**Supplementary Figure 1:** Process of enrollment, categorization of study subjects

Among the 834 patients, 12 patients were excluded due to insufficient data or advanced stage. Finally, 922 patients were divided into two groups according to the CONUT score.

CONUT, controlling nutritional status
